# Does the Biosocial Model Explain the Emergence of Status Differences in Conversations among Unacquainted Men?

**DOI:** 10.1371/journal.pone.0142941

**Published:** 2015-11-20

**Authors:** Allan Mazur, Keith M. Welker, Bin Peng

**Affiliations:** 1 Maxwell School, Syracuse University, Syracuse, NY, 13244, United States of America; 2 Psychology Department, University of Massachusetts Boston, Boston, MA, 02125, United States of America; 3 Economics Department, Syracuse University, Syracuse, NY, 13244, United States of America; Brock University, CANADA

## Abstract

Fifteen triads of unacquainted men conversed for ten minutes while stress was measured in real time by pulse rate and thumb blood volume (TBV). Salivary measures of testosterone (T), cortisol (C), and the stress-related enzyme alpha-amylase (AA) were measured at the beginning and end of the session. Fully or partially transitive status hierarchies formed in 14 triads. (Highest ranked man was scored 1, lowest 3, with ties allowed.) Ten of the triads participated in Study 1, where nothing was at stake in the casual conversation. Five additional triads were run in Study 2, intended to introduce competition by offering a $20 reward to the man afterward chosen as having led the conversation. Most results from the two studies are similar, suggesting that the $20 reward had little effect. Combining studies, pulse and TBV show that conversation is more stressful than watching a video beforehand. Within the conversation, speaking turns are more stressful than listening turns, especially among the lowest ranked men, less so among those higher in rank. This supports a stress-based mechanism for status allocation among humans. Apparently, human speech is a form of status signaling, homologous with nonlinguistic status signals used by other primates, as posited by the “biosocial model.” The biosocial model also posits that a physiological substrate (T, C, and AA) is related to dominance or status. Predicted effects are not replicated here, except for an inverse relationship between the stress enzyme AA and status. The mostly null results, obtained from conversations where there was little or nothing at stake, suggest that T and C (and their interaction) are not relevant to emergent status in the absence of serious competition.

## Introduction

A classical finding in small group research is that previously unacquainted subjects brought together into discussion groups usually sort themselves into status hierarchies, with some acting as leaders, others as followers. Often this happens quickly, even when subjects show no obvious status cues (or “diffuse status characteristics”) that differentiate them at the outset [1, 2]. A person’s position in the status hierarchy is measured by numerous correlated indicators: amount of speech, ability to introduce or end topics of discussion, the extent to which one is the focus of attention, nonverbal demeanor including body posture and facial expressions, and via evaluations by group members of leadership and quality of participation [3–7].

Traditional theories of status and leadership in face-to-face groups, following for example Bales [8] and Homans [9], treat humans as sui generis and assume that their allocation of status in primary groups is mediated by human-level cognition. If the physical body is relevant at all, it is through external appearance (e.g., race, size, gender, age) or, as in Goffman [10], by postures and nonverbal gestures of assertion or deference. In contrast, a newer “biosocial model” emphasizes that status processes in face-to-face human groups are not unique but follow a general primate pattern and that, as among other primates, physiology is highly reactive during social interaction, affecting and being affected by status allocation.

Despite considerable empirical work on the biosocial model in recent decades, two central questions remain unanswered and are the subject of this paper. Nearly all research has been on overtly competitive, strongly goal-oriented activities including games and athletic contests. *Here we ask if the previously identified physiology of status is also important in casual conversations among unacquainted men when nothing is at stake*. The second question concerns the centrality of language for humans. Theorists have the choice of treating our species as unique, to be explained on our own terms as in the tradition of Bales and Homans, or of treating language as simply one of several modes of signaling whereby primates communicate status-relevant information to conspecifics, as in the biosocial model. Taking the latter course, *we ask if human speech works physiologically like other dominant and deferent signs used throughout the primate order*, *specifically in affecting the stress levels of interlocutors*.

These questions are addressed in two studies relating physiology to the emergence of status during conversation. Study 1 is of ten triads of unacquainted men engaged in casual free discussion with nothing at stake. Pulse rate and thumb blood volume (TBV) were measured as real-time indicators of stress. The hormones testosterone (T) and cortisol (C) and the enzyme alpha-amylase (AA) were measured from saliva samples taken near the beginning and end of the session. For comparison, five additional triads were run in Study 2 where competition was introduced by offering a $20 reward to the man afterward voted as having led the discussion.

## The Biosocial Model

Dominance (or status) hierarchies are a reliable feature of face-to-face primate groups. Status rank may be persistently relevant in species with fairly permanent groups, or only occasionally relevant for animals that forage alone. Rankings are usually, but not necessarily, transitive. Low-ranked members often appear more nervous than higher-ranked members; high-ranked members can manipulate the stress experienced by–and thereby the performance of–low-ranked members (see [4] for an overview).

Two similar biosocial models associate testosterone with dominant behavior, one formulated by Mazur [11] for primates, the other by Wingfield et al. [12] for birds. In the primate model, every individual has certain observable signs or signals that suggest his or her social status is (or ought to be) high or low. High or rising T is presumed to support the expression of high-status signs, while low or declining T shifts signaling toward deferent signs. Dominant signaling can induce stress in the interaction; deferent signaling reduces stress.

Visualize two individuals (Ego and Alter) meeting for the first time. If their interaction is very brief or casual, the notion of ranking may never arise. In more extended or serious meetings, each appraises the status signs of the other, forming some idea of their relative standing. If Ego perceives that Alter’s status signs exceed his own, he may defer to Alter. Among humans, Ego may explain that Alter *belongs* in the higher rank, or that Alter *deserves* it, or that Alter *could easily take it* if Ego resisted, or that Alter may be more *competent* in the duties of high rank. If neither Ego nor Alter accepts the low rank, they may compete, each producing dominant signals that induce stress until one switches to a deferent mode (or withdraws), thus relieving his felt stress and in so doing accepting lower status. Such exchanges may be violent, e.g., among macaques, but that is rare among apes and especially humans, who usually form their status hierarchies in a polite and friendly manner, avoiding overt competition or, when that does occur, barely aware of it if the stresses are at a low level.

Ego’s decision to comply or compete depends on his motivation to dominate and the stakes on the table. When Ego is on home territory, or protecting group members or valued possessions, and Alter is an intruder, then Ego is particularly likely to rise to a challenge. Among humans, a substantive disagreement–perhaps over a point of information or ideology–may escalate into a dominance competition so that winning becomes an end in itself, with the original substantive disagreement relegated to secondary importance.

In the biosocial model, conversation is simply one mode of signaling. Dominant or deferent signs may be communicated in what is said (“I came, I saw, I conquered” vs. “I am the dust beneath your feet”), in postures and gestures that accompany speech, in amount of speaking time (i.e., holding or yielding the floor), in setting or accepting topics for discussion or otherwise directing the conversation, and in opening or closing the interaction [4]. Here we examine empirically the simplest kind of conversational dominance/deference, i.e., whether an individual is speaking or listening. It is this difference that explains the generally reliable finding that those of high status speak more than those of low status [3].

## Physiological Substrate

Testosterone is associated with dominant, assertive, and leader-like behavior, at least among males in serious competition. The link between T and dominance is reciprocal. Not only does T affect dominance behavior, but changes in dominance behavior or in social status cause changes in T in competitions as varied as athletic events, laboratory tasks, market transactions, and elections. However there are caveats: T effects are most often found among subjects who compete seriously, when a considerable reward or their reputation is at stake. Also T level per se may not be as important in predicting dominant behavior as changes in T in anticipation of serious competition [13–23]. These caveats render uncertain the role, if any, that T plays in casual conversational groups where little or nothing is at stake.

Cortisol, a product of the hypothalamus-pituitary axis, is often called the “stress hormone” because it usually elevates under physically or socially stressful conditions (see review in [24]). According to the “dual hormone hypothesis,” T is associated with dominance or leadership especially when C is low. This hypothesis is intuitively appealing, suggesting that the people most likely to act assertively, to break norms or otherwise scale stressful barriers, are those least bothered by the stressor, thus low in C. The import of a TxC interaction for dominant behavior is now an active area of research, with studies to date often but not always affirmative (e.g. [25–28]).

Acute stress activates the hypothalamic-pituitary-adrenal (HPA) axis and sympathetic nervous system (SNS), producing elevations in the salivary enzyme alpha-amylase (AA) as well as in C and heart rate. Cortisol and AA might rise together in some stressful situations, but having different physiological bases, they need not act in concert. C is a hormone originating in an endocrine gland of the HPA axis, circulating in the blood; AA is not, being produced in the exocrine salivary glands of the SNS. The diurnal pattern of AA shows a steep decline within 30 minutes of waking followed by graduate increase through the day, virtually the opposite of C’s diurnal pattern [29]. C and AA are best considered alternate responses to stress [30].

Recently AA has been proposed as a sensitive biomarker for activity specifically of the sympathetic nervous system in response to psychological (and physical) stress [31]. That C and AA tap different aspects of the stress response is illustrated by their differing reactions to three laboratory methods for inducing psychological stress. For example, Ss were given the Trier Social Stress Test, which includes public speaking in front of a panel of judges, and their C levels elevated immediately afterward, remaining high for a half hour, while AA was unresponsive [32]. On the other hand, AA responds to other stressors [33].

Like T and C, AA is conveniently measured through saliva, and it is being increasingly used in psychological experimentation. To date, AA has not been used in research on the biosocial model of status, but because of its emerging importance to studies of stress, it is included in this inquiry.

**Fig 1:**
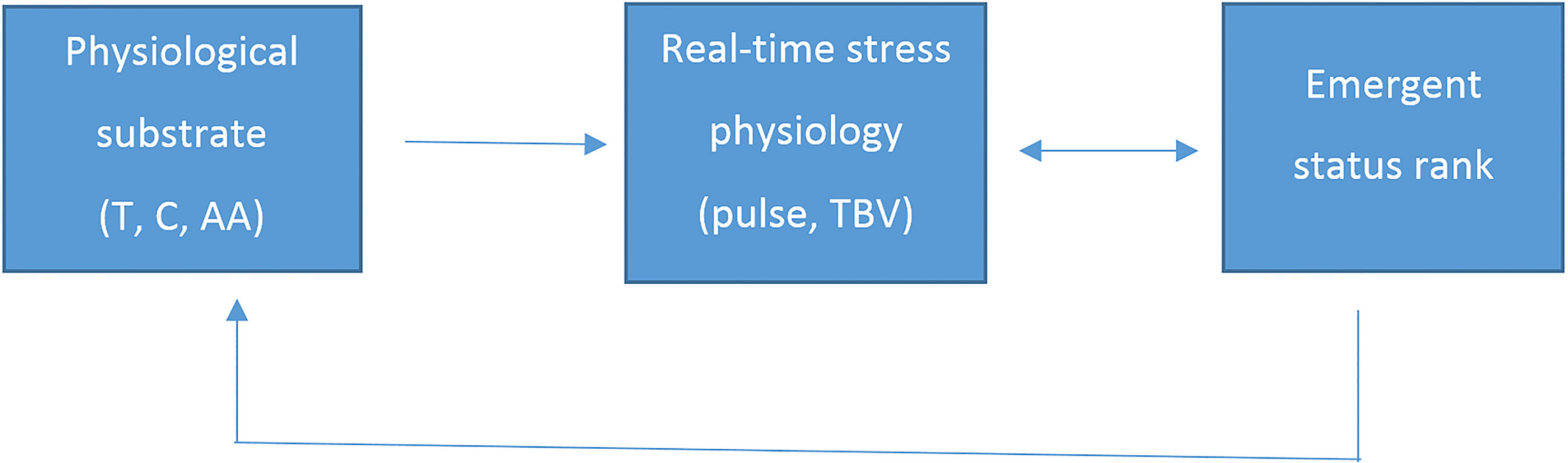
The biosocial model.

Research on human subjects consistently shows a time lag (usually on the order of minutes) between the elevation of a hormone and its related behavior. In contrast, peripheral physiological measures, specifically pulse rate and thumb blood volume, respond almost instantly, permitting the monitoring of a subject’s stress level in real time. The biosocial model can take advantage of these differently timed responses, placing the slower-reacting substrate of hormones and alpha-amylase temporally (and perhaps causally) prior to instantaneous (real-time) changes in pulse and TBV. The biosocial model is conceptualized in Fig 1 where emergent status feeds back to both real-time physiology and the prior substrate.

## Methods

### Participants and Procedure

All procedures for this study were approved by the Institutional Review Board of Syracuse University. All subjects provided written consent. The experimenter (E), a senior professor who held sessions in his office/laboratory, dressed casually and acted informally. Unacquainted subjects (Ss), run in groups of three, were randomly assigned chairs at a rectangular table, arranged so their conversation could be videoed. One man was seated at the left end of the table (from the perspective of the video camera), and two sat beside one another on a long side, facing the camera.

Males from the university community (excluding steroid users) were recruited through campus advertisement, offered $20 for participation in an afternoon session of approximately forty minutes during which saliva samples would be requested for assay. Of 45 Ss, 36 were American (31 white, 5 black), eight South Asian, and one East Asian. All had adequate conversational fluency. Nine Ss were graduate students or alumni, 36 were undergraduates. Ages ranged from 18 to 26 years old. When possible, Ss were scheduled to avoid important variation in external (“diffuse”) status characteristics, such as mixing graduate and undergraduate students, old and young, or having one foreign student the conspicuous minority in a triad. Given constraints on subject availability, this was not always achievable.

At the outset, E paid each S $20, explained the study, and obtained informed consent. Ss gave their first (“prior”) saliva sample. E attached a photoplethysmographic sensor to the fleshy side of one thumb of each S, and then, to allow some settling of physiological response, played a relaxing video, the 200-second flight sequence from *Out of Africa* (1985 Academy Award winner for best picture). This famous scene shows the romantically involved costars in a biplane flight over scenic Africa. Then E explained,

*I’d like you to talk to each other for about ten minutes about any topics of your choosing*. *I know from experience that the most popular topics are sports*, *music*, *travel*, *or school*, *but the conversation can be about anything*. *OK*, *go ahead*.

Nothing was at stake in these conversations. Study 2 was intended to create more competitive discussions. Its procedure differed from Study 1 only in this addition to E’s instruction:

*Sometimes one man tends to lead the conversation*, *the others following*. *Should that happen*, *I’ll give that person another $20*. *You will decide at the end of the discussion if there was such a leader*. *If there is a tie*, *I’ll be the tie-breaker*.

Ss entered free discussion for ten minutes while E remained in the room, away from the table. Afterward E removed the thumb sensors and asked Ss to complete a questionnaire rating each person’s contribution of ideas and effectiveness in leading the discussion. Then Ss gave their post-conversation saliva samples. Ss were debriefed and excused.

In Study 2, after the final saliva collection, E awarded $20 to the man who received most votes on the questionnaire items asking who most effectively led the discussion, and who contributed the best ideas. E never had to break a tie.

### Physiological Variables

#### Saliva substrate

Ss had been instructed via email to avoid alcohol, recreational drugs and excessive caffeine on the day of the study. Sessions ran in the afternoon between 1:00 and 5:00 to reduce diurnal variation in physiology. Saliva collection followed standard procedures [31, 33, 34]. Ss flushed their mouths with water before drooling or spitting unstimulated saliva into a collection tube, giving about two mL in roughly two minutes. After completing the post-discussion questionnaire, Ss gave a second (post) sample about five minutes after the conversation and about twenty minutes after the prior sample. For five triads in each study, a third saliva sample was collected five minutes later to explore whether a time delay would provide more pronounced hormonal changes, however this sample provided no noteworthy information and is not discussed further. All samples were placed in frozen storage.

Frozen saliva was sent to Salimetrics at Arizona State University where T and C were assayed using commercially available enzyme immunoassays. For C the range of sensitivity was .007 to 3.0 μg/dL; for T the range of sensitivity was from 1.0 to 600 pg/mL. Salimetrics reports inter-assay and intra-assay precision (coefficient of variation) for both assays, on average, of less than 15 percent and 10 percent respectively. Duplicate assays for this study gave CVs for T and C of < 5 percent.

The decision to include salivary alpha-amylase as a biomarker was not made until saliva from the first five triads of Study 1 had been assayed and destroyed. Salimetrics measures AA using a reliable kinetic reaction assay with only one step (no antibody is involved, see [31]), so tests are not duplicated. From a prior study, Salimetrics calculated an intra-assay coefficient of variation for AA of less than 8 percent, and inter-assay variation of less than 6 percent. Expressed in units of activity per milliliter (U/mL), AA can range from undetectable to more than 2,000 U/mL.

In this study, T varies from 52 to 351 pg/dL (mean = 134, median = 123); C from 0.06 to 0.48 μg/dL (mean = 0.18, median = 0.16); and AA from 9 to 413 U/mL (mean = 83, median = 57). All values are in normal ranges. As usual, distributions of T and AA have positive skew and are transformed to lnT and lnAA. C has a slight positive skew not improved by transformation and is left untransformed. Pairwise correlations: r = 0.12 for lnT and C, r = 0.15 for lnT and lnAA, and r = 0.13 for C and lnAA.

#### Real-time measures

A photoplethysmographic sensor was connected by Velcro cuff to S’s thumb so with each pulse beat an electric signal is sent to a computer. The resulting waveform moves up and down with the beating heart. TBV and pulse rate are recorded continuously through a Biopac MP35 interface using BSL 4.0 software, enabling signals from thumb sensors to be integrated with the video recording for simultaneous playback. The software is versatile for making calculations.

Changing TBV is measured by the changing distance from the top of one beat to the bottom of the next (referred to as “peak-to-peak value”). As blood flows away from the thumb (under stress), the waveform narrows; as blood flows back to the thumb (relaxation), the waveform widens [35–37].

Ss often change sitting position, or wave or tap their Velcro-cuffed thumbs on the table, introducing noise into the waveform, usually briefly or at modest levels that can be ignored. For four Ss, these perturbations were severe, warranting correction. For one fidgety S, 15 seconds of noise were excised from the waveform. E interrupted a second S, asking that he hold his hand still; immediately the amplitude of that S’s waveform showed a step-like increase. A third S had a step-like increase in amplitude immediately after changing posture, and a fourth S showed a step-like increase after waving his cuffed hand to make a point. These step-like increases were eliminated (i.e., statistically adjusted) based on the ratio of mean amplitudes 20 seconds before and 20 second after the break.

An advantage of pulse rate, measured as beats per minute (BPM), is that it is directly interpretable to assess changes over time within a subject, or differences between subjects.

Raw values of peak-to-peak TBV are partly a function of the tightness of the Velcro cuff on S’s thumb, so these are not interpretable until normalized. For example, change in TBV from watching *Out of Africa* to conversation is calculated by taking the ratio: mean peak-to-peak during the conversation/mean peak-to-peak during the flying sequence. Another normalization that will be used here calculates the ratio: mean peak-to-peak during an S’s speaking turns/mean peak-to-peak during his listening turns.

### Status and Leadership Variables

The *aggregated* status ranks of group members were assessed by combining seven component measures: gestalt perceptions of the status hierarchy by three judges who independently watched the videoed discussions; counts from the videos of amount of speaking time, number of topics introduced to the discussion, and number of speaking turns; and combined ratings by Ss in each triad of who among their triad mates most effectively led the discussion and contributed the best ideas. For each component, status rank is rated from 1 (high) to 3 (low). When a pair of Ss is not consistently differentiated on at least four of these seven component measures, that pair is counted a tie (each is scored 1.5 if tied for top, 2.5 if tied for bottom). There are eight ties out of a possible 45 pairings.

The three judges who independently made gestalt evaluations of status rank were a conversational analyst, a layperson, and E. (The first two judges were blind to other data.) The judges’ ratings are in high agreement, pairwise correlations running from r = 0.74 to 0.91 (combining studies). As expected, the seven component measures of status were redundant, pairwise correlations running from r = .23 to 0.91 (median r = .59). The pattern of correlations is similar in both studies except that Ss’ evaluations of their triad mates’ contributions to the discussion, which matching other status indicators in Study 1, lost construct validity in Study 2. For example, in Study 1, Ss’ evaluations were highly correlated to aggregate status rank (r = 0.90), but in Study 2 this correlation dropped to r = 0.19. Apparently in Study 2, where a $20 reward was at stake for the man judged leader, Ss were gaming their evaluations.

Videoed conversations include many quick utterances, periods of short verbal exchange, listeners briefly questioning or commenting on the speaker, and periods of silence. A speaking turn is defined as a fairly consistent utterance by one person lasting more than four seconds (allowing short pauses or interjections by others).

Data are incomplete for two triads in Study 1. One man asked permission to leave before completing his questionnaire and was excused without providing post-conversation saliva. Another man’s waveform was unusable because of inexplicable noise that obscured his pulse signal. Data upon which this analysis is based appear in S1 Table.

### Statistical Analysis

The group-based design of this study violates the assumption of standard OLS regression that for each combination of values of the independent variables, the outcome observations are independent. If one man in the triad holds top rank, ranks of the other two men are constrained; thus status rank has non-independence within triads [38]. To correct for this violation of interdependence, we used multilevel modeling [39] to test the main effects and interactive effects of T, C, and AA on status rank. Using the SPSS MIXED procedure, identifiers of group member number and group number were taken as repeated and subject variables, respectively. All multilevel analyses used a diagonal covariance structure.

Specifically, the biosocial model suggests that high status will be achieved by men with high T, or low C (or low AA), or the combination of high T and low C. To test these hypotheses, interaction effects were computed from mean-centered predictor data. The patterns of the interaction effects were decomposed using the tools developed by Preacher, Curran, and Bauer [40] for simple slopes analysis in multilevel models. These tools tested the simple slopes of predictors at high and low values of the specified moderators (±1 SDs) using procedures recommended by Aiken and West [41]. Interaction effects are plotted using scatterplots, with a median split conducted on the moderator. Median splits are provided for ease of viewing the data but were not used in the formal tests of interaction effects and simple slopes.

## Results

### Status in the Discussion

The aggregated measure of status/leadership showed status structuring in all but one of fifteen triads (S2 Table). In Study 1 (triads A through J), eight groups had transitive 1-2-3 hierarchies, and two groups had no differentiation between the two highest ranking men (given rank 1.5), but the bottom rank was clear. In Study 2 (triads M through Q), two groups had transitive 1-2-3 hierarchies; two groups had no differentiation between the two highest ranked men, but the bottom rank was clear; and one triad had no discernible hierarchy (all men given rank 2). The greater ambiguity of ranking in Study 2 suggests more competition for status, but with only five triads that conclusion is not firm.

Diffuse status characteristics are known to affect status ranking, an effect minimized here by trying to match Ss within each triad. Among four triads composed of two undergraduates and one (older) graduate, the graduate attained top rank twice. In two triads composed of two white Americans and one foreign or racial minority, the minority had bottom rank once. Overall, diffuse status characteristics played a minor role and are hereafter ignored.

Ss occupying the rightmost seat (video camera’s perspective) were at a disadvantage in status ranking. Entering a dummy for seating position produced no important effects on physiological relationships, so seating position is hereafter ignored.

### Real-Time Stress (Pulse and TBV) Changes with Conversation and Status

Laboratory Ss in dyads showed higher stress during conversation than in a preceding or following quiet period [36, 42]. During the quiet period here, triads watched the flying sequence from *Out of Africa*. If a man were wholly relaxed, the waveform measured from a thumb sensor would undulate smoothly at the frequency of his resting pulse, its amplitude constant, a reflection of regular heartbeat and unconstricted blood vessels in the thumb. Ss’ actually recorded waveforms (as well as E’s subjective impression) attest that complete relaxation was virtually never achieved while sitting at the lab table, even under the lulling influence of *Out of Africa*. Mean pulse rate during the flying sequence was 77 BPM (combining both studies), accelerating to a mean of 84 BPM during the ten-minute conversations (p < .001, paired comparison t-test, one tail). Mean TBV, normalized at 1.00 during the flying sequence, shrunk to 0.77 during conversation (p = .001, paired comparison t-test, one tail). Thus, reports of higher stress during conversation in dyads were replicated here for triads.

Thirty-two men (combining studies) had *both* faster pulse rate *and* lower TBV during the discussion than when watching *Out of Africa*. (Two men showed the opposite pattern.) The combination occurs in 73 percent of Ss in each study. Often the visual appearance of the waveform, as it is traced out on the screen, clearly marked the transition point in the session. There was a distinct compression of amplitude as TBV constricted (and pulse quickened) with the onset of talking.

The biosocial model goes further, predicting variation in stress *within* the discussion, higher while conversing dominantly, lower while conversing deferentially (homologous to dominance/deference signaling in other primates). In simplest terms, taking the floor in a discussion is an assertive action; attentively listening is deferential. It should follow that speaking turns are more stressful than listening turns.

The range of speaking turns (of at least four seconds duration) across all triads is 3 to 13 turns. Mean TBV and pulse rate are measured while each man speaks and while he listens (i.e., when others are speaking). The ratio of TBV-while-speaking to TBV-while-listening provides normalized TBV. Stress during speaking (relative to listening) is indicated by a TBV ratio < 1. The actual mean ratio = 0.88 (combining studies, p = .002, t-test, one tail). There is no significant difference between studies, the mean ratio in Study 1 = .87, in Study 2 = .89. The ratio is < 1 in 79 percent of Ss in Study 1 and 87 percent of Ss in Study 2 (ns).

If speaking is more stressful than listening, the ratio of pulse rate during speaking turns, to pulse rate during listening turns, should be > 1. The actual mean ratio = 1.09 (combining studies, p < .001, t-test, one-tail). There is no significant difference between studies, the mean ratio in Study 1 = 1.07, in Study 2 = 1.12. The ratio is > 1 in 93 percent of Ss in Study 1 and 87 percent of Ss in Study 2 (ns). Thus, both TBV and pulse rate show more stress during speaking turns than during listening turns.

**Fig 2:**
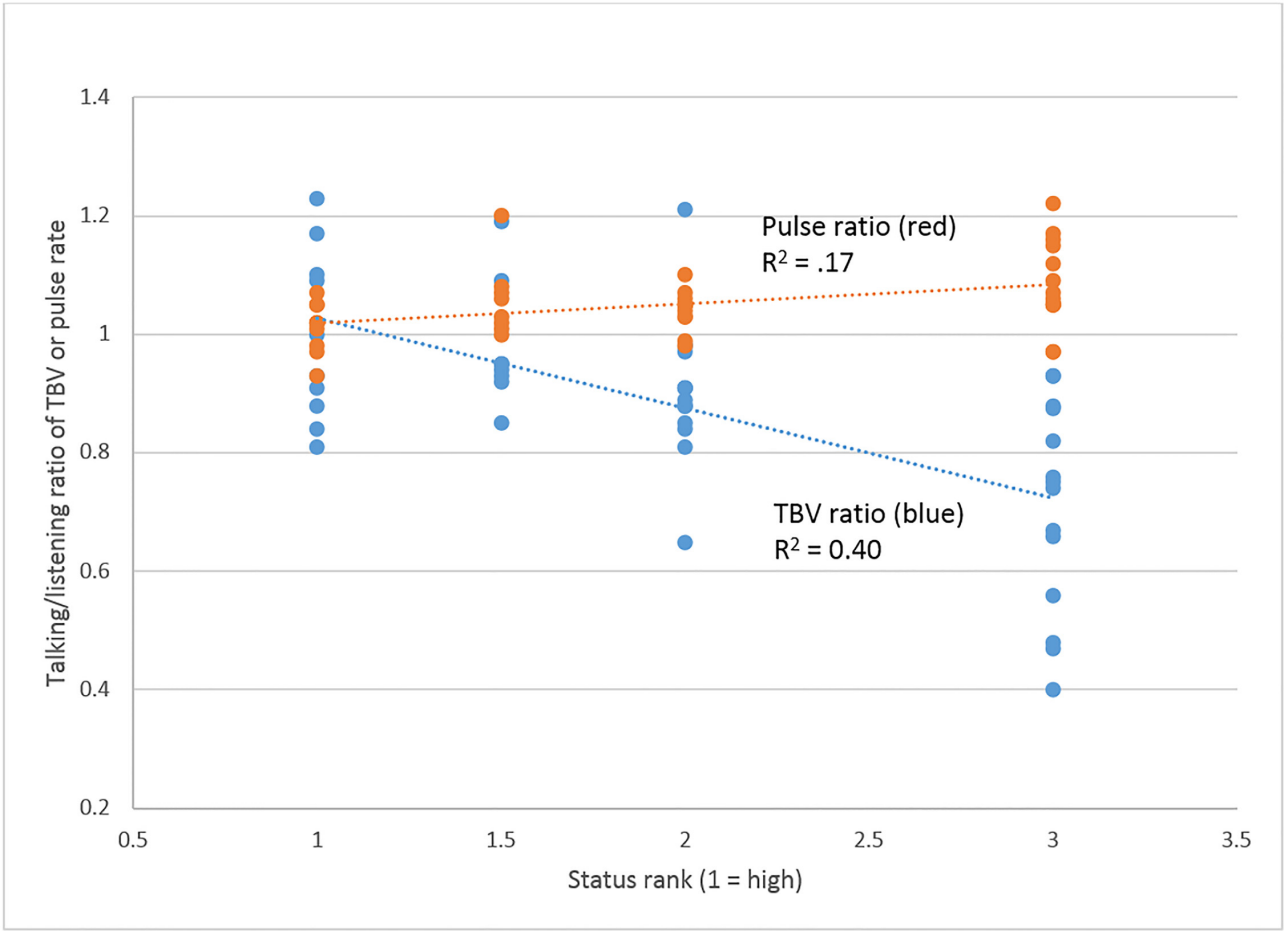
Talking/listening ratio of TBV is higher, and of pulse rate is lower, among high-ranked men (combining studies).

According to the biosocial model, high status goes to those who most comfortably manage, or are least affected by, the stress of dominance interaction. I.e., the stress of speaking (relative to listening) should be less for high-ranked men than for low-ranked men. Combining studies, Fig 2 shows status rank on the horizontal axis (1 = high status, 3 = low status), and speaking-to-listening ratios for TBV and pulse on the vertical axis. As predicted, the ratio for TBV slopes downward, and the ratio for pulse rate slopes upward, as status rank descends (both p < .001). TBV ratio has the stronger effect.

### The Physiological Substrate (T, C, and AA) and Status

The biosocial model expects hormones to react to status dynamics. In the present studies, means of lnT, C, and lnAA barely change from prior to post-conversation saliva samples (Table 1). In Study 1, the mean absolute value of change from prior ln T to post lnT = 0.21; mean absolute value of change from prior C to post C = 0.04, and the mean absolute value of change from prior lnAA to post lnAA = 0.95. In Study 2, the corresponding changes are 0.19, 0.07, and 0.44. Thus, changes in hormones and AA from before to after the conversations do not greatly exceed the sensitivities of the assays (see Methods). Combining studies, correlations between prior and post values are r = 0.69 for lnT, r = 0.77 for C, and r = 0.44 for lnAA. Overall, lnT and C are fairly stable across the session, whereas lnAA was less stable.

**Table 1:** Mean LnT, C, and LnAA from prior and post saliva samples*.

	Prior lnT	Post lnT	Prior C (μg/dL	Post C (μg/dL)	Prior lnAA	Post lnAA
Study 1	4.77 (n = 30)	4.82 (n = 29)	0.19 (n = 30)	0.19 (n = 29)	4.33 (n = 15)	4.43 (n = 15)
Study 2	4.91 (n = 15)	4.89 (n = 15)	0.18 μg/dL (n = 15)	0.17 μg/dL (n = 15)	3.67 (n = 15))	3.84 (n = 15)

*Differences from prior to post are not significant by paired-comparison t-tests. Post and prior lnAA are significantly lower in Study 2 than in Study 1 (t-tests); values of lnT and C are not.

The $20 reward notwithstanding, there are no significant differences in lnT or C between Study 1 and Study 2. Unexpectedly and inexplicably, prior and post values of lnAA are significantly *lower* in Study 2 than Study 1 (p ≤ .05, t-tests), a difference that cannot be attributed to the manipulation because Ss did not know of the $20 reward until after the prior saliva was collected.

Going further, we used 3-level multilevel models to predict hormones, with time of saliva collection (prior vs. post) nested within individuals, which were in turn nested in triads. In these models, we used whether the $20 reward was present or not (Study 1 vs. Study 2), Status Rank, and Time as mean centered predictors, along with all possible interaction terms. Three separate models were conducted, with lnT, C, and lnAA as outcomes (Table 2). Among these three models, only two main effects emerge as significant. First, there is a main effect of study number on lnAA (B = -.63, p = .01), consistent with higher lnAA in Study 1 than in Study 2, already noted in Table 1. Second, there is a main effect of status rank on lnAA (B = .29, p = .04), indicating that lower ranking men had higher AA. Neither lnT nor C is significantly related to status (though the weak relationship of status rank to C approaches significance).

**Table 2:** Hormones and AA as functions of competition (Study 1 vs Study 2), time, and status rank.

Outcomes:	lnT		Cortisol		lnAA	
Predictor	B (SE)	p	B (SE)	p	B (SE)	p
Competition	0.10 (.08)	0.21	-0.01 (.02)	0.51	-0.63 (.20)	0.01
Time	0.01 (.08)	0.87	0.00 (.02)	0.92	0.16 (.21)	0.45
Status rank	0.08 (.05)	0.13	-0.03 (.01)	0.06	0.29 (.14)	0.04
Competition X Time	-0.06 (.16)	0.70	-0.01 (.05)	0.76	-0.01 (.40)	0.98
Competition X Rank	0.06 (.11)	0.60	0.05 (.03)	0.10	0.34 (.28)	0.23
Time X Rank	0.01 (.10)	0.89	0.02 (.03)	0.50	-0.03 (.28)	0.91
Time X Rank X Comp.	0.31 (.23)	0.19	0.05 (.06)	0.48	0.17 (.55)	0.76

Since there is little change in hormones or AA from prior to post saliva samples, the next analyses are based on mean levels. (Separate analyses for prior or post values produce similar results.)

In the biosocial model, T is associated with dominant or high status behavior. The “dual hormone” hypothesis further suggests that T affects dominance/status especially in people who have low C. This is tested by regressing status rank on lnT, C, and an interaction term for lnT by C. This interaction term is routinely obtained by first centering variables in the interaction on zero, then multiplying them together [43].

Since AA also is indicative of stress, we assessed an interaction term for lnT*lnAA in a second model. (AA was measured in only five triads of Study 1, so there is more statistical power in the lnT*C model for Study 1.) Our multilevel models, displayed in Table 3, were run separately for Study 1 and Study 2.

**Table 3:** Multilevel models of status rank as a function of lnT, C, and lnAA in Studies 1 and 2.

Study 1					Study 2				
	*B*	*SE*	*t(df)*	*p*		*B*	*SE*	*t(df)*	*p*
LnT	0.35	0.38	.93 (21.82)	0.36	LnT	0.637035	0.84	.76 (5.23)	0.48
C	-1.68	1.23	-1.37 (23.89)	0.19	C	-0.56967	3.50	-.16 (9.84)	0.88
LnT X C	7.72	3.95	1.96 (24.70)	0.06	LnT X C	10.01957	21.9	.46 (8.80)	0.66
	*B*	*SE*	*t(df)*	*p*		*B*	*SE*	*t(df)*	*p*
LnT	1.72	0.34	5.10 (1.67)	0.05	LnT	0.03	0.56	.06 (6.34)	0.95
LnAA	0.07	0.17	.44 (2.78)	0.69	LnAA	0.67	0.29	2.33 (7.89)	0.05
LnT X LnAA	-3.48	0.84	-4.12 (1.63)	0.08	LnT X LnAA	0.18	0.90	.20 (9.47)	0.84

**Fig 3:**
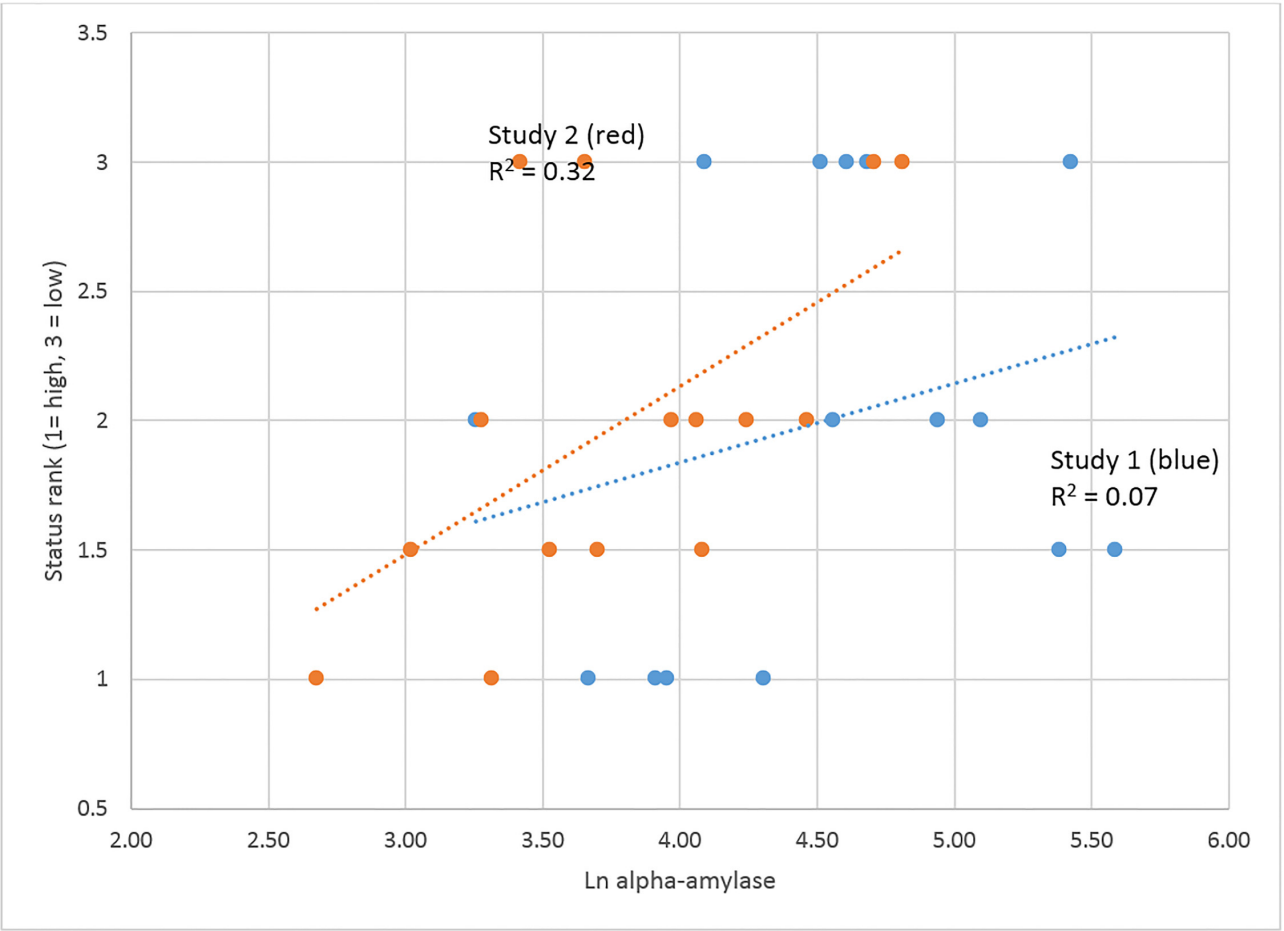
High alpha-amylase is associated with low status (shown for both studies).

Across both studies, lnT and C are not associated with status rank in the predicted directions. LnAA is significantly associated with status rank in Study 2 indicating, as expected, higher stress among lower ranked men in this more competitive situation. The AA-status relationship is plotted in Fig 3, separately for the two studies, illustrating the stronger association in Study 2.

Testing the dual hormone hypothesis, there was a marginally significant LnT x C interaction in Study 1. Decomposing this interaction revealed that lnT was associated with lower rank when C was high (+1SDs: B = 1.19, t(24.70) = 2.28, p = .03) but not low (-1 SDs: B = -.49, t(24.70) = .80, p = .44), a pattern not consistent with the dual hormone hypothesis.

In Study 1, there was also a marginally significant LnT X LnAA interaction (*B* = -3.48, *p* = .08). The pattern of this interaction suggests that T was associated with lower status when AA was low (-1SDs: *B* = 4.09, *t*(1.63) = 6.26, *p* = .020), but not high (+1SDs: *B* = -.65, *t*(1.63) = .95, *p* = .402). If we regard AA as analogous to C in the dual hormone hypothesis, i.e., both indicators of stress, then again the pattern is inconsistent with the hypothesis.

Overall, the only verified effect of the physiological substrate that is expected from the biosocial model is the significant association of lower status with higher AA (Study 2).

### Dynamic Changes in Hormones Predicting Rank

Although we did not find substantial changes in lnT, C, or lnAA across the conversations, we still investigated whether their slight changes predict rank during the interaction. These changes were calculated by subtracting prior lnT, C, and lnAA from the corresponding post levels. The prior-to-post changes were used as individual predictors of status rank in single multilevel models for Studies 1 and 2. Results are presented in Table 4. No hormonal or AA changes were associated with status rank in the non-competitive Study 1 (all ps ≥ .47). However, in Study 2 with the $20 reward, increases in lnT (B = .012, t(5.02) = 3.19, p = .02) and C (B = 4.58, t(10.56) = 2.57, p = .03) were significantly associated with lower rank, their positive slopes indicating slightly higher values at lower status rank. In addition, lnAA changes in Study 2 had a nearly significant association (B = .014, t(7.69) = 2.16, p = .07), with slightly increased AA at lower rank.

**Table 4:** Relationships of status rank to prior-to-post changes in lnT, C, and lnAA.

	lnT Changes		C Changes		lnAA Changes	
	B (SE)	p	B (SE)	p	B (SE)	p
Study 1 (Non-competitive)	-.001 (.003)	.72	1.94 (2.61)	.47	.001 (.001)	.51
Study 2 (Competitive)	.012 (.004)	.02	4.58 (1.78)	.03	.014 (.006)	.07

### The Physiological Substrate ((T, C, and AA) and Real-Time Physiology (Pulse and TBV)

In prior sections we saw that real-time measures of stress (pulse, TBV) perform as expected during conversation. We now turn to links between the hormones or enzyme, on the one hand, and pulse and TBV, on the other. Stated concisely, lnT, C, and lnAA have no significant, consistent and substantial relations to pulse and TBV under the present conditions.

### Power Analysis

Limited by funding, the numbers of triads in these studies were small. The unexpected failure to show significant T (or interaction) effects on status, even after an element of competition was added in Study 2, leads us to ask if there was insufficient statistical power for relationships to be detected even if they were actually present. In other words, given our small samples, we may have failed to reject the null hypothesis even though we should have.

The *power* of a test is the probability of correctly rejecting the null hypothesis when it is false, or in other words, the likelihood of identifying a significant effect when one exists. Obviously, the larger the sample, the higher the power. Conventionally, a power of .80 or higher is desirable.

To illustrate this concept, we conducted post hoc power analyses for the joint F-test of the four standardized regressions in Table 2 for OLS regressions, setting alpha = 0.05. Although we formally used multilevel modeling to test these analyses, we present power analyses using OLS for two primary reasons. First, power analysis for multilevel modeling has not been extensively developed and varies depending on the sample size at Levels 1 and 2 [44, 45]. Second, researchers have primarily conducted dual hormone interaction effects with the more commonly-used moderated regression analysis [25, 46]. We observe the R^2^ of the four models to be 0.22, 0.51, 0.09, and 0.34, which represent median, large, small, and large effect sizes respectively, according to Cohen’s standards [47]. The statistical powers for the four regressions are 0.61, 0.47, 0.12 and 0.81, respectively. Note that the very low power (0.12) of the third regression (Model A for Study 2) for detecting a small effect means that if small relations between hormones and status do actually exist, we have almost a 90 percent chance of observing a non-significant result, so our failure to find significant effects in this model is not compelling. On the other hand, we also failed to find these effects in Model A for Study 1, which has more respectable power (0.61). And, since these two models are independent of one another, their combined failure to find significant effects is weightier still. (Obviously, larger hormone effects would be more detectable.)

## Discussion

The biosocial model pictures status hierarchies of primate groups as arising from an exchange of dominant (or deferential) signals that during competition raise (or lower) stress [4]. Leadership goes to individuals most willing and able to accommodate such stresses, while those more stress-averse occupy subordinate positions.

Applying the model to humans requires that language, our most important medium of communication, fulfills the function of status signaling, raising and lowering stress. During a discussion, taking the floor is an assertion of prerogative, a dominant signal, while silently listening is deferential. Accordingly, speaking should be more stressful than listening. Is this true?

The answer is yes. In both studies, pulse rate and TBV show higher stress during conversation than when watching a video. More importantly, within the conversation, speaking turns are more stressful than listening turns. Still more importantly, degree of stress varies with status: Higher-ranked men are more at ease speaking to the group than are lower-ranked men (Fig 2). Probably as a consequence, high status members typically monopolize group discussions [3].

Since talking requires more energy than listening, one might inquire if energy expenditure alone, rather than stress, explains the different cardiovascular responses while speaking than while listening to a speaker. If true, one would also have to assume that during their speech turns, higher status men expend less energy than lower status men; otherwise the observed relationship between status and cardiovascular response is inexplicable. One would also expect that listening during the conversation would have the same pulse and TBV responses as listening during the video, which we did not observe.

Apart from real-time stress measures (pulse, TBV), the biosocial model presumes that T influences status attainment during serious competition. Does this occur as well in casual conversations with little at stake? Our answer is no, though this is provisional considering the modest sample sizes. Possibly a T effect would have emerged after a longer conversation, or if post-conversation saliva sampling had been delayed, however there was no sign of it in the third saliva sample, taken 25 minutes after the first saliva, which in this laboratory has been sufficient time to detect a response. In any case, in the present study high T did not predict high status nor was it raised by achieving high status. Even under modest competition, when adding a $20 reward for the man rated as leader, T had no positive relationship to status rank, directly or in interaction.

The one substrate effect that did occur was that men with lower stress, as measured by AA, attained higher rank in Study 2 when $20 was at stake; the relationship was slight without the reward (Fig 3). The link holds for prior- as well as post-conversation measures of AA, so if there is a causal connection, it likely runs from being initially relaxed to attaining high rank in the conversation. (AA may be more sensitive than C as an indicator of stress.)

The dual hormone hypothesis is an intuitively appealing modification of the biosocial model, proposing that T affects dominance only when actors are not inhibited by stress, as measured by C being low [25]. The enzyme alpha-amylase is used here as an alternative indicator of stress, so the same reasoning holds [31]. Such moderator effects, whether interactions of T and C or of T and AA, failed to significantly predict conversational status in the expected direction.

Obviously there is a gap between literature reporting direct (or moderated) T effects during serious competition, and no effect here when competition is absent or slight. (The $20 reward seems not to have added much competition.) These differing results are not contradictory but do emphasize that T effects probably become observable only during high stakes competition. High stakes need not be monetary and could involve personal reputation or matters of symbolic importance. A desirable study for the future would again involve conversational groups but with more variation in the importance of competition, from low to high. The biosocial model predicts that T effects will appear as rewards for leadership increase.

The biosocial model posits that higher stress is associated with lower status, a relationship that is absent here when C is the measure of stress. However, as noted above, when AA is the measure of stress, the expected inverse relationship to status does occur, especially in Study 2 when an element of competition was introduced (Fig 3). Bearing in mind the different physiological bases of C and AA, the former a product of the HPA axis, the latter of the sympathetic nervous system, we propose that sympathetic nervous activity is first to come on line, at low levels of stress, and that the HPA axis will not be activated until stress reaches a moderate or higher level. All of our findings must be regarded as tentative because of small sample sizes, but if these results are sustained in future studies, the biosocial model should incorporate the notion that very casual or unimportant interactions produce little if any stress and little involvement of the physiological substrate. As the seriousness of the interaction increases, first the sympathetic nervous system is activated, and only with a further increase in seriousness does the HPA axis and more generally the physiological substrate come into play.

A reality of research life is the constraint imposed by limited funding. We could afford to run relatively few triads and assays. Perhaps compensating for the small numbers, we introduce potentially useful innovations into research on the biosocial model. While C is the traditional workhorse measure for psychological stress, salivary AA has only recently come on the scene [31]. Ours is the first research to use AA with the biosocial model, and it related more strongly to status than did C, at least among conversational triads.

Perhaps our most important innovation is the use of pulse and TBV for real-time measurement of stresses during status interaction. Simultaneous playback of these cardiovascular tracks along with a video of the conversation makes visible an otherwise hidden dimension of group process, showing when one member has an increase in stress, when another becomes more relaxed, and so on. These changes are instantaneous compared to the minutes-long response times of T, C and AA, and more sensitive, enabling a “microscopic” view of bodily reactions as people sort themselves into ranks of the hierarchy.

## Supporting Information

S1 Table Data table. (XLSX)

S2 Table Final status ranking in each triad combines rankings based on judges’ gestalt status ranks, quantitative measures of the discussion, and subjects’ own evaluations. (DOCX)
