# Supplementary material for: Does the Biosocial Model Explain the Emergence of Status Differences in Conversations among Unacquainted Men?
Source: PLoS One. 2015 Nov 20;10(11):e0142941. doi: 10.1371/journal.pone.0142941 (PMC4654577; doi:10.1371/journal.pone.0142941)
Supplement: S2 Table — (DOCX) [file pone.0142941.s002.docx]

Final status ranking in each triad combines rankings based on judges’ gestalt status ranks, quantitative measures of the discussion, and subjects’ own evaluations.

|  | **Triad A** | **Triad B** | **Triad C** | **Triad D** | **Triad E** |
| --- | --- | --- | --- | --- | --- |
| **Final**  **rank** | Judges Time Turns  Topics Self | Judges Time Turns  Topics Self | Judges Time Turns  Topics Self | Judges Time Turns  Topics Self | Judges Time Turns  Topics Self |
| **1** | C:1,1,1 1 1  1 1 | C:1,1,1 1 1.5  1.5 1.5 | L:1,1,1 1 1  2 1 |  | L: 1,1,2 1 1  2 1 |
| **1.5** |  |  |  | R:1,1,2 3 1.5  2 1  C:1,2,2, 1 1.5  1 2 |  |
| **2** | R:2,2,2 2 2  2 2 | R:2,2,2 2 1.5  1.5 1.5 | C:2,2,2 2 3  3 2.5 |  | C:1,2,2 2 2  1 2 |
| **2.5** |  |  |  |  |  |
| **3** | L:3,3,3 3 3  3 3 | L:3,3,3 3 3  3 3 | R:3,3,3, 3 2  2 2.5 | L:3,3,3 2 3  3 3 | R:3,3,3 3 3  3 3 |

* Subjects’ seating positions Left, Center and Right, are indicated as L, C and R. ”Judges” indicates the gestalt status rankings of three judges. “Time” is status rank by speaking time. “Turns” is status rank by number of speaking turns longer than four seconds. “Topics” is rank according to number of topics set. “Self” is rank according to Ss’ own evaluations of who most effectively led the discussion and who contributed the best ideas.

Table A1 (continued).

|  | **Triad F** | **Triad G** | **Triad H** | **Triad I** | **Triad J** |
| --- | --- | --- | --- | --- | --- |
| **Final**  **rank** | Judges Time Turns  Topics Self | Judges Time Turns  Topics Self | Judges Time Turns  Topics Self | Judges Time Turns  Topics Self | Judges Time Turns  Topics Self |
| **1** | C:1,1,1 1 1  2 2 | L:1,1,1 1 1  1 1 |  | L:1,1,1 2 1  1 1 | R:1,1,1 2 2   1. 1 |
| **1.5** |  |  | R:1,1,2 2 2.5   1. 2   C:2,2,1 1 1  3 1 |  |  |
| **2** | L:2,2,2 2 3  1 1 | R:2,2,2 3 2  2 2 |  | C:2,2,2, 1 2  2 2 | C:2,2,2 1 1  2 2 |
| **2.5** |  |  |  |  |  |
| **3** | R:3,3,3 3 2  3 3 | C:3,3,3 2 3  3 3 | L:3,3,3 3 2.5  2 3 | R:3,3,3 1 3  3 3 | L:3,3,3 3 3  3 3 |

Table A1 (continued).

|  | **Triad M** | **Triad N** | **Triad O** | **Triad P** | **Triad Q** |
| --- | --- | --- | --- | --- | --- |
| **Final**  **rank** | Judges Time Turns  Topics Self | Judges Time Turns  Topics Self | Judges Time Turns  Topics Self | Judges Time Turns  Topics Self | Judges Time Turns  Topics Self |
| **1** | C:1,1,1 1 1  1 2 |  |  |  | L:1,1,1 1 1  1 1 |
| **1.5** |  | C:1,1.5,2 2 1.5   1. 1   L:1,1.5,2 1 1.5   1. 3 | R:1,1.5,2 1 1  2 2  C:1.5,2,2.5 2 2  1 3 |  |  |
| **2** | L:2,2,2 3 3   1. 1 |  |  | R:1,2,3 3 3   1. 1   C:1,2,3 1 2   1. 2.5   L:2,2,2 2 1  3 2.5 | C:2,2,2 2 2.5  2 2 |
| **2.5** |  |  |  |  |  |
| **3** | R:3,3,3 2 2  3 3 | R:3,3,3 3 3  3 2 | L:2,2.5,3 3 3  3 1 |  | R:3,3,3 3 2.5  3 3 |
